# Salivary Glands Proteins Expression of *Anopheles dirus* A Fed on *Plasmodium vivax-* and *Plasmodium falciparum*-Infected Human Blood

**DOI:** 10.1155/2013/535267

**Published:** 2013-07-15

**Authors:** Saowanee Cotama, Paron Dekumyoy, Yudthana Samung, Usa Lek-Uthai

**Affiliations:** ^1^Program in Infectious Diseases and Epidemiology, Faculty of Graduate Studies, Mahidol University, Nakornpathom 73170, Thailand; ^2^Department of Parasitology and Entomology, Faculty of Public Health, Mahidol University, Rajvithi Road, Bangkok 10400, Thailand; ^3^Department of Helminthology, Faculty of Tropical Medicine, Mahidol University, Rajvithi Road, Bangkok 10400, Thailand; ^4^Department of Medical Entomology, Faculty of Tropical Medicine, Mahidol University, Rajvithi Road, Bangkok 10400, Thailand

## Abstract

Mosquitoes are able to adapt to feed on blood by the salivary glands which created a protein that works against the haemostasis process. This study aims to investigate the salivary glands proteins expression of 50 adult female *An. dirus * A mosquitoes, a main vector of malaria in Thailand, each group with an age of 5 days which were artificial membrane fed on sugar, normal blood, blood infected with *P. vivax*, and blood infected with *P. falciparum*. Then mosquito salivary gland proteins were analyzed by SDS-PAGE on days 0, 1, 2, 3, and 4 after feeding. The findings revealed that the major salivary glands proteins had molecular weights of 62, 58, 43, 36, 33, 30, and 18 kDa. One protein band of approximately 13 kDa was found in normal blood and blood infected with *P. vivax* fed on day 0. A stronger protein band, 65 kDa, was expressed from the salivary glands of mosquitoes fed with *P. vivax*- or *P. falciparum*-infected blood on only day 0, but none on days 1 to 4. The study shows that salivary glands proteins expression of *An. dirus * may affect the malaria parasite life cycle and the ability of mosquitoes to transmit malaria parasites in post-24-hour disappearance observation.

## 1. Introduction 

Malaria is a mosquito-borne infectious disease and a major public health problem in the world. Malaria is widespread in tropical and subtropical regions, including much of sub-Saharan Africa, Asia, and the Americas [1]. The spread of malaria is often associated with environmental and mosquito vector factors, and the vectors of malaria are female *Anopheles* species. In Thailand, there are 3 major vector species, *An. dirus*, *An. Minimus*, and *An. maculatus* [2], of which *An. dirus* is the most common. It has been found that *An. dirus* is the major carrier of malaria in Thailand [3, 4]. Adult mosquito salivary glands are paired organs located on either side of the thorax flanking the esophagus [5, 6]. The female gland has three lobes, including two lateral lobes, with distinct proximal and distal portions, and a medial lobe. Salivary glands contain several kinds of protein [7], but female glands contain approximately 10 times more protein than male glands. Each lobe of the female mosquito salivary gland differs in protein contents, with the distal lobe having the most proteins [5]. The rate of protein accumulation in the salivary glands is highest on day 2 of growth as adult, reaching a peak in week 2 and then beginnings to decline at week 3 [8]. *Anopheles *spp. must feed on human or animal blood and need nutrients in the blood to stimulate growth of ovaries and encourage eggs creation. The female mosquito requirement for blood has made this an opportunity for infective malaria sporozoites that live in salivary glands to be transmitted to humans or animals when the female mosquito takes a blood meal by an infected mosquito bites. Mosquitoes feed on blood as quickly as possible to avoid haemostasis processes, consisting of platelet aggregation, vasoconstriction, and blood coagulation. However, the platelet inhibitors, vasodilators, and anticoagulation protein in mosquito saliva inhibit haemostasis allowing the sporozoite stage of malaria to spread the disease through their frequeny biting activity [9, 10]. In this study the salivary glands proteins expression of *An. dirus* A fed on sugar, normal blood, blood infected with *P. vivax*, and blood infected with *P. falciparum* was examined. 

## 2. Materials and Methods

### 2.1. Malaria Parasites

A group of 200 five-day-old *Anopheles dirus* A (*An. dirus*) females from the same generation were divided into 4 groups of 50 in each group and fed on sugar, normal blood, patients' blood infected with *P. vivax,* and blood infected with *P. falciparum*, respectively. The Certificate of Approval Ethical Review Committee for Human Research, Faculty of Public Health, Mahidol University, COA no. MUPH2011-213 was approved.

### 2.2. Mosquito Rearing

*An. dirus* mosquitoes were maintained in an insectary of the Department of Medical Entomology, Faculty of Tropical Medicine, Mahidol University, Thailand. Conditions were set at 27 ± 2°C and 70 ± 10% relative humidity under 12/12 hrs light/dark photoperiod. Adult mosquitoes were given continuous access to a 10% sugar solution.

### 2.3. Artificial Membrane Feeding

This study used normal blood from volunteers, blood of patients infected with malaria from Hospital for Tropical Diseases, Mahidol University. Normal blood and patient's blood were drawn by vacutainer with K3 EDTA. Mosquitoes were fasted overnight, the feeding time was 30 min–1 hour, and then all fed mosquitoes were maintained in a controlled chamber of 27°C and 70 ± 10% humidity, under a 12 hrs light/dark cycle and fed with sugar-soaked cotton wools. 

### 2.4. Mosquito Salivary Gland Dissection

Mosquitoes were anesthetized with ether, and salivary glands were dissected on days 0, 1, 2, 3, and 4 after feeding using fine entomological needles under a stereomicroscope in 1x phosphate buffered saline (PBS) and then transferred into a microcentrifuge tube containing 10 *μ*L of ice cold PBS solution and stored at −80°C prior to SDS-PAGE.

### 2.5. Sodium Dodecyl Sulphate Polyacrylamide Gel Electrophoresis (SDS-PAGE)

Samples of 10 pairs of female salivary gland were individually heated in SDS loading buffer at 95°C for ten minutes and loaded on 12% SDS polyacrylamide gels according to the method of Laemmli [11]: 0.5 M Tris-HCl buffer: 12.12 g Tris-HCl, pH 6.8, 8 mL 10% SDS, and 110 mL H_2_O (stock 4x stacking gel Tris); 1.5 M Tris-HCl buffer: 36.40 g Tris-HCl, pH 8.8, 8 mL 10% SDS. For the preparation of the separating and stacking solutions were performed (5.25 mL H_2_O, 6 mL of 30% stock acrylamide solution (30 g Acrylamide, 0.8 g Bis-acrylamide, and 100 mL H_2_O), 3.75 mL of 1.5 M Tris-HCl buffer, 150 *μ*L of 10% ammonium peroxodisulfate (0.1 g ammonium peroxodisulfate, 1 mL H_2_O, and 15 *μ*L TEMED). Molecular mass markers (Thermo Scientific) were applied in each gel.

### 2.6. Coomassie Brilliant Blue (CBB) Staining

Following electrophoresis, gels were CBB-stained (1.25 g Coomassie blue R250, 250 mL absolute methanol, 50 mL glacial acetic acid, and 500 mL distilled water) for 2 hrs on the overhead shaker at room temperature. Thereby, the gel is equally stained blue. To make the proteins visible, the gel has subsequently to be stained in destaining solution (225 mL absolute methanol, 50 mL glacial methanol, 25 mL glycerol, and 750 mL distilled water) which the process has to be changed several times until the background is relatively colorless until dark protein bands appear. 

## 3. Result

### 3.1. The Morphology of the Salivary Glands of Adult Female *An. dirus*

**Figure 1:**
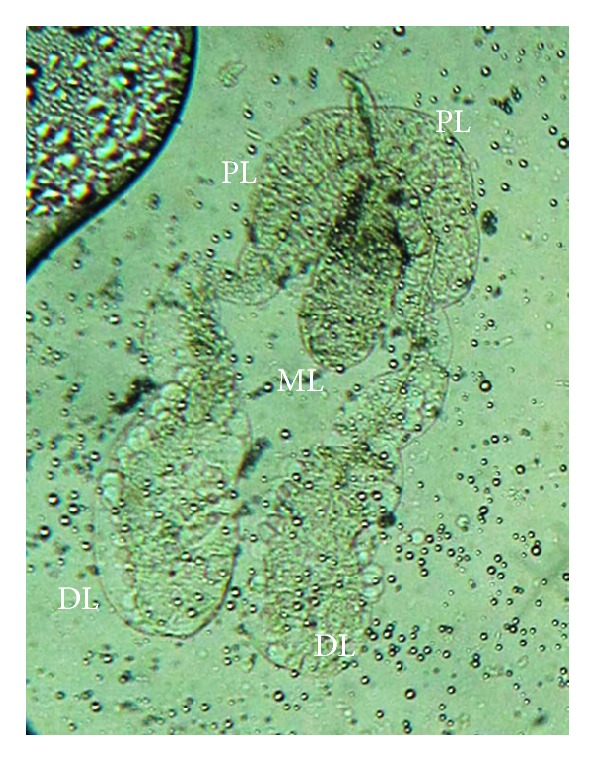
Adult female salivary glands of *An. dirus*. PL: proximal region of lateral lobe; DL: distal region of lateral lobe; ML: median lobe.

The salivary glands of adult *An. dirus* were paired organs lying in the thorax on either side of the esophagus. The female gland was composed of two identical lateral lobes and a single median lobe. The lateral lobe of each female salivary gland was composed of two secretory regions, proximal and distal, while the medial lobe has only one region (Figure 1).

### 3.2. SDS-PAGE Analysis of the Proteins Present in the Salivary Glands of *An. dirus*

**Figure 2:**
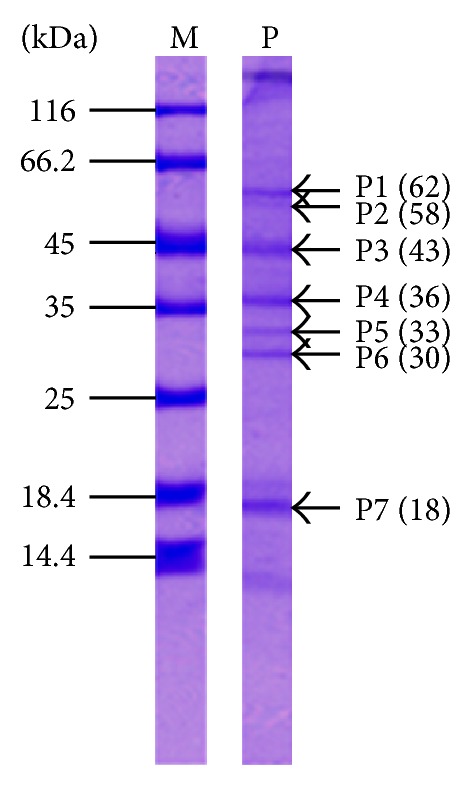
Salivary gland proteins of female *An. dirus* mosquitoes were separated on 12% SDS polyacrylamide gels and stained with CBB-stained. Labels on the right indicate major protein bands (P1−P7) and their estimated molecular weights (in blankets). Lane M: protein standard markers (kDa); lane P: salivary glands proteins.

The total proteins present in the salivary glands of female *An. dirus* at day 5 after emergences were examined by SDS-PAGE and CBB. There were at least 7 major and several minor protein bands. Major protein bands with estimated molecular mass were 62, 58, 43, 36, 33, 30, and 18 kDa. A few protein bands were produced at the lowest part (<14.4 kDa, standard marker) of the gel (Figure 2). 

### 3.3. SDS-PAGE Analysis of the Protein Profile in Mosquitoes at Days 0, 1, 2, 3, and 4 after Feeding on Sugar, Normal Blood, Blood Infected with *P. vivax*, and Blood Infected with *P. falciparum*

**Figure 3:**
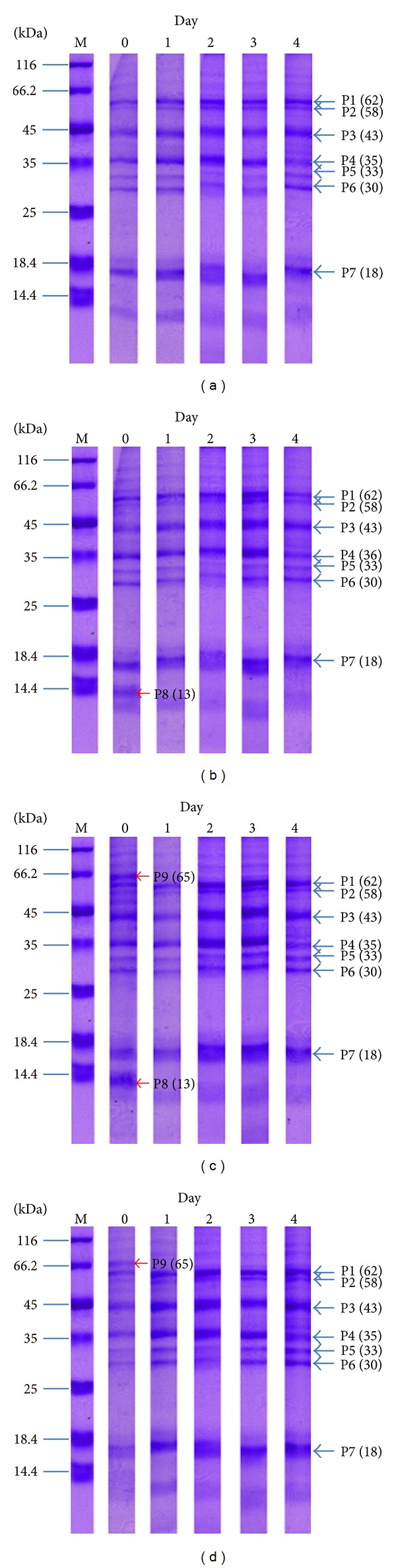
The proteins profiles of the salivary glands was followed up on days 0, 1, 2, 3, and 4 after mosquitoes feeding on sugar (a), normal blood (b), *P. vivax*-infected blood (c), *P. falciparum*-infected blood (d). Their molecular weights were presented on SDS polyacrylamide gels with CBB stain.

 The expression patterns of proteins in the salivary glands of mosquitoes fed on different meals were examined. The proteins profiles of the salivary glands were followedup on days 0, 1, 2, 3, and 4 after mosquitoes had fed on sugar, normal blood, *P. vivax*-infected blood, *P. falciparum*-infected blood. The molecular masses of the major bands were 62, 58, 43, 36, 33, 30 and 18 kDa. The protein profiles of the salivary gland of sugar-fed mosquitoes extracts on different days showed similar profiles that presented 7 major protein bands and weak and big bands at <14.4 kDa (Figure 3(a)). In the normal blood fed mosquito group, a 65 kDa protein appeared as a small band on day 0. A minor band (<30 kDa) obviously appeared by days 2 and 3 and reduced by day 4. An estimated 13 kDa protein (P8) appeared predominantly at day 0 only (Figure 3(b)). The proteins present in the salivary glands of mosquitoes fed on *P. vivax*-infected blood also showed molecular masses of 62, 58, 43, 36, 33, 30, and 18 kDa. Additionally, two bands estimated at 13 and 65 kDa appeared predominantly at day 0 only. A minor band (<30 kDa) obviously appeared by days 2 (Figure 3(c)). The proteins profiles of the salivary glands from mosquitoes fed on *P. falciparum*-infected blood showed similar profiles that presented 7 major protein bands and a minor band (<30 kDa) obviously that appeared by days 2. The estimated 65 kDa protein band appeared predominantly at day 0 but a 13 kDa band did not appear in salivary glands of *P. falciparum*-infected blood fed mosquitoes (Figure 3(d)).

### 3.4. The Intensity Value of Salivary Glands Proteins from Sugar-, Normal Blood-, *P. vivax*-Infected Blood-, and *P. falciparum*-Infected Blood- Fed Female Salivary Glands *An. dirus* A

**Figure 4:**
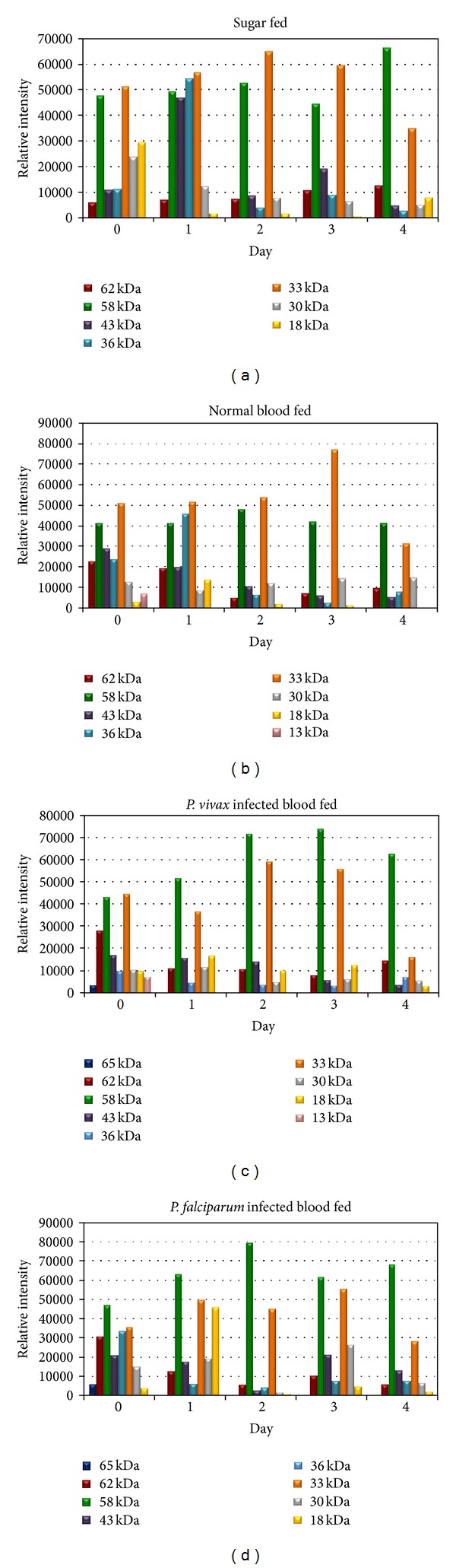
Relative profiles of the protein bands at 65, 62, 58, 43, 36, 33, 30 and 18 kDa from sugar (a), normal blood (b), *P. vivax* infected blood (c), *P. falciparum* infected blood (d) fed female salivary glands *An. dirus*. The bar graph indicated the relative intensity after their infected blood feeding at days 0, 1, 2, 3 and 4.

 The relative intensity values of salivary glands of 58 and 33 kDa proteins bands in sugar feeding group were higher than other proteins bands (62, 43, 36, 30 and 18 kDa proteins) in every day (0–4 days). In addition the intensity values of 43 and 35 kDa proteins were high at day 1 after sugar-feeding (Figure 4(a)), while the 36 kDa protein had high intensity value at day 1 in normal blood feeding group. The 13 kDa protein appeared at day 0 only, when compared to other proteins bands (62, 58, 43, 36, 33, 30, and 18 kDa proteins) (Figure 4(b)). The 58 and 33 kDa proteins in *P. vivax*-infected blood feeding group were higher intensity value than other proteins bands in every day. The 13 and 65 kDa proteins appeared at day 0 were lower in intensity when compared to other proteins bands (62, 58, 43, 36, 33, 30, and 18 kDa proteins) (Figure 4(c)). However, after *P. falciparum* infected-blood feeding group, the 58 and 33 kDa proteins were higher intensity value other proteins bands in every day, in the 18 kDa protein was high intensity value at day 1 after feeding. The 13 kDa protein appeared at day 0 were lower when compared to intensity values when compared to other proteins bands (62, 58, 43, 36, 33, 30, and 18 kDa proteins) (Figure 4(d)).

## 4. Discussion

### 4.1. The Morphology of Salivary Glands of Female *An. dirus* A

The morphology of *An. dirus* A salivary glands is similar to other mosquito species previously reported, *Armigeres subalbatus* [7], *An. dirus* B (or *An. scanloni*) [5], *An. darlingi* [12], *Aedes togoi* [13], and *Mansonia uniformis* [14]. The salivary glands of female *An. dirus* are composed of two identical lateral lobes and a medial lobe. However, the lateral lobes can be divided into proximal and distal regions. 

### 4.2. Proteins Electrophoresis Pattern of Salivary Gland of Adult Female *An. dirus* A after Emergence

 In this study, the overall profiles of female salivary gland proteins of *An. dirus* A were analyzed. The protein profiles contained at least seven major proteins and several minor proteins visualized after SDS-PAGE. The proteins profiles of *An. dirus* A (*An. dirus*) in this study were similar to those of the pattern from *An. dirus* B (*An. scanloni*) with molecular masses of 63, 44, 43, 35, 33, 30, and 18 kDa. However, there is a different result between major protein bands at 44/43 kDa, as only the 43 kDa component was found in this study. Also, a 58 kDa band is clearly seen in our study, but it is not mentioned in study of Jariyapan et al. [5]. In this study, the lowest part (<14.4 kDa, standard marker) of the protein pattern was variable in the early days of feeding, days 0–3, where it seems to be weak or disappeared in all feeding groups. 

### 4.3. Protein and Intensity Value of Salivary Glands Proteins Profiles Compared with Sugar Fed, Normal Blood, and Blood Infected with Malaria Parasites Fed

 Comparing those mosquitoes fed on normal blood, blood infected with *P. vivax*, and blood infected with *P. falciparum*, it was found that the expression of different proteins and intensity value in salivary glands was detected. At day 0 of the mosquito fed on blood containing malarial parasites, the 65 kDa protein clearly appeared and was seen as a small band in the normal blood feeding group and was not seen in the sugar feeding group. This may indicate that *P. vivax* and *P. falciparum* malarial parasites may be involved in increasing the amounts of this protein. The 65 kDa protein was immediately produced (after infected blood feeding and then dissection of salivary glands) and disappeared in a short time. It was also unseen on days 1–4. This protein and the intensity value should have continued to be studied during its duration of disappearance in 24 hours, according to the unseen protein in post-24-hour observation (day 1). 

 An observation of 30 kDa proteins was found in a sharp band on day 0 of four feeding groups, and then a minor band (<30 kDa) gradually increased on days 1-2 and continued decreasing on days 3-4. It became a sharp band on day 4. This development may have occurred from carbohydrate metabolism due to the protein which was not seen on day 0 in all feedings. This protein and intensity value began increasing from day 1 with more content on day 2. It started to reduce from day 3 onwards and became a sharp band in all groups on day 4. Its development differs from the 65 kDa protein of feeding groups with *P. vivax*-infected blood and *P. falciparum*-infected blood, which did not happen in the sugar feeding group. This evidence also occurs in the development of 16 kDa. However, it seems to be more involved with blood content rather than carbohydrate content in the sugar feeding group because 16 kDa is not seen in the sugar feeding group on day 0 but is seen in three blood feeding groups on day 0. The 16 kDa was seen in all groups from days 1–4. However, this protein was reported to reduce at a faster rate in the sugar group than the other three groups on day 4.

In addition, a 13 kDa protein and intensity value appear in groups of normal blood-fed and *P. vivax*-infected blood-fed mosquitoes, not in sugar-fed and *P. falciparum*-infected blood-fed groups. This protein is also increased when compared with the normal blood-fed group. If any content in normal blood is involved in stimulating production of this protein, it is strange that 13 kDa is unseen in the *P. falciparum*-infected blood-fed group. It is interesting that such content of *P. vivax* malaria stimulates the 13 kDa protein expression in salivary glands, which is not found in the *P. falciparum*-infected blood-fed group. Another further study is required on different content between *P. vivax* and *P. falciparum* malaria. In the particular proteins in other studies, these proteins and intensity value have responsibilities or feeding functions. Suwan et al. [15] reported that approximately 36 kDa bands in *An. stephensi* were characterized and identified as a member of the D7 salivary protein family. Specifically, it was found in median lobe and distal lateral lobes. The D7 protein plays a crucial role in the blood feeding process [16, 17]. A study on the salivary gland proteins of sugar and normal blood-fed *An. gambiae* mosquitoes, used protein profiles of these 2 groups compared after 1 hour feeding. The latter salivary glands show marked high and low molecular mass proteins. The most notable difference seen is the expression of the 100 kDa protein in response to a blood meal in mosquitoes. The expression of the 29 kDa salivary gland protein changed a little in response to a blood meal and is comparable to that of sugar-fed mosquitoes [18].

In other mosquito species from previous reports, Siriyasatien et al. [7] studied total salivary gland proteins of *Ar. subalbatus* mosquitoes at 0, 6, 24, and 48 hours after a blood meal. The results revealed that the 65 and 21 kDa protein bands, immediately after blood feeding, were detected, but both proteins started to appear gradually 6 hours later and returned to the unfed level in 48 hours. Phumee et al. [14] studied female *Ma. uniformis* salivary gland proteins of 37 kDa, which gradually decreased after a blood meal, whereas proteins and intensity value of 61 and 83 kDa started to increase dramatically during 12 to 24 hours after feeding. The salivary glands proteins profile of female *An. darling* showed similar profiles of the proteins expression between blood-fed and sugar-fed mosquitoes in different times. Moreira et al. [19] suggested that there was a specific protein to be induced by blood content. Jariyapan et al. [5] reported that the major protein bands in the glands of both sugar-fed and blood-fed *An. dirus* B showed similar protein profiles after a followup. In a comparative study between the different species above, *An. darlingi* and *An. dirus* B, the protein profiles of salivary glands with normal blood-fed and sugar-fed groups show no difference. The protein profiles of *Ar. subalbatus* and *Ma. uniformis* reveal that proteins increase and decrease dramatically after feeding. The protein profiles of *An. gambiae* salivary glands are changed when the mosquitoes are dissected immediately after normal blood feeding. Then, 1 hour after normal blood feeding, protein profiles were similar to the sugar-fed group. The salivary glands protein profiles of *An. gambiae* are correlated to our study after immediately normal blood feeding and sugar feeding. The astonishing findings are *An. dirus* B (*An. scanloni*) and *An. dirus* A (*An. dirus*) that the same as dirus complexes group, but protein profiles of both species are comparable in the difference between sugar-fed and normal blood fed mosquitoes. The salivary glands protein profile of *An. dirus* change after normal blood feeding, not in *An. dirus* B. However, the results of different protein and intensity value profiles blood-fed and malaria infected blood-fed may be the basis for future studies of the role of protein in salivary glands of *An. dirus* mosquitos that affect the malaria parasite life cycle development and the ability to transmit the malaria parasite.
